# Leveraging Large Language Models for Simulated Psychotherapy Client Interactions: Development and Usability Study of Client101

**DOI:** 10.2196/68056

**Published:** 2025-07-31

**Authors:** Daniel Cabrera Lozoya, Mike Conway, Edoardo Sebastiano De Duro, Simon D'Alfonso

**Affiliations:** 1 School of Computing and Information Systems, The University of Melbourne, Gratham St, Parkville VIC, Melbourne, 3010, Australia, 61 90355511; 2 Department of Psychology and Cognitive Science, University of Trento, Trento, Italy

**Keywords:** medical education, mental health, chatbots, psychotherapy training, virtual client

## Abstract

**Background:**

In recent years, large language models (LLMs) have shown a remarkable ability to generate human-like text. One potential application of this capability is using LLMs to simulate clients in a mental health context. This research presents the development and evaluation of Client101, a web conversational platform featuring LLM-driven chatbots designed to simulate mental health clients.

**Objective:**

We aim to develop and test a web-based conversational psychotherapy training tool designed to closely resemble clients with mental health issues.

**Methods:**

We used GPT-4 and prompt engineering techniques to develop chatbots that simulate realistic client conversations. Two chatbots were created based on clinical vignette cases: one representing a person with depression and the other, a person with generalized anxiety disorder. A total of 16 mental health professionals were instructed to conduct single sessions with the chatbots using a cognitive behavioral therapy framework; a total of 15 sessions with the anxiety chatbot and 14 with the depression chatbot were completed. After each session, participants completed a 19-question survey assessing the chatbot’s ability to simulate the mental health condition and its potential as a training tool. Additionally, we used the LIWC (Linguistic Inquiry and Word Count) tool to analyze the psycholinguistic features of the chatbot conversations related to anxiety and depression. These features were compared to those in a set of webchat psychotherapy sessions with human clients—42 sessions related to anxiety and 47 related to depression—using an independent samples *t* test.

**Results:**

Participants’ survey responses were predominantly positive regarding the chatbots’ realism and portrayal of mental health conditions. For instance, 93% (14/15) considered that the chatbot provided a coherent and convincing narrative typical of someone with an anxiety condition. The statistical analysis of LIWC psycholinguistic features revealed significant differences between chatbot and human therapy transcripts for 3 of 8 anxiety-related features: negations (*t*_56_=4.03, *P*=.001), family (*t*_56_=–8.62, *P*=.001), and negative emotions (*t*_56_=–3.91, *P*=.002). The remaining 5 features—sadness, personal pronouns, present focus, social, and anger—did not show significant differences. For depression-related features, 4 of 9 showed significant differences: negative emotions (*t*_60_=–3.84, *P*=.003), feeling (*t*_60_=–6.40, *P*<.001), health (*t*_60_=–4.13, *P*=.001), and illness (*t*_60_=–5.52, *P*<.001). The other 5 features—sadness, anxiety, mental, first-person pronouns, and discrepancy—did not show statistically significant differences.

**Conclusions:**

This research underscores both the strengths and limitations of using GPT-4-powered chatbots as tools for psychotherapy training. Participant feedback suggests that the chatbots effectively portray mental health conditions and are generally perceived as valuable training aids. However, differences in specific psycholinguistic features suggest targeted areas for enhancement, helping refine Client101’s effectiveness as a tool for training mental health professionals.

## Introduction

### Background

Psychotherapy training requires a comprehensive approach, encompassing practical skill development through supervised sessions with clients or peer role-playing, along with the analysis and discussion of therapy sessions from experienced psychologists [1]. Optimal psychotherapy training ideally needs abundant practice opportunities coupled with immediate performance-based feedback [2]. However, both training methods, working with clients or using role-play, present significant challenges. The use of clients raises ethical concerns regarding patient welfare, particularly when inexperienced psychotherapists provide treatment, which can result in risks to vulnerable individuals receiving suboptimal care. However, role-play scenarios, while more controlled, are resource-intensive. They face challenges such as finding suitable peers for role-playing and ensuring a consistent learning experience due to varying skill levels among participants.

The integration of natural language processing (NLP) in the field of mental health training offers potential solutions to challenges in client availability. Chatbots leveraging large language models (LLMs) can simulate human dialogue and offer a structured framework for task-oriented interactions [3]. Their evolving conversational abilities allow them to actively engage in dialogues, making them a promising educational tool in fields such as health care and medical education [4]. The use of chatbots as virtual patients in mental health training holds significant potential. Through prompt engineering techniques, chatbots can be configured to simulate diverse mental health conditions and behaviors. This capability makes them valuable assets for psychotherapy training in a controlled environment, thereby mitigating the risks associated with using real clients for training purposes. Additionally, chatbots offer the advantage of being readily accessible platforms for simulated therapeutic interactions at any time.

In this study, we present Client101, a web-based conversational platform that uses LLMs as chatbots to simulate the behavior of mental health clients. We used 2 distinct prompt configurations to generate 2 types of virtual clients: one simulating the experience of depression and the other representing individuals coping with generalized anxiety disorder (GAD). A total of 16 individuals with a background in psychotherapy (ie, clinical psychologists and qualified counselors) used the platform to evaluate it. The participant therapists were tasked with conducting single sessions with each of the 2 chatbots before completing a questionnaire. While no specific instructions were given, it was suggested to the participants that they use something like a single-session integrated cognitive behavioral therapy (SSI-CBT) approach [5].

Furthermore, we used the LIWC-22 (Linguistic Inquiry Word Count) software to measure psycholinguistic features of the sessions. This enabled us to examine whether the chatbots used linguistic indicators commonly associated with depression and GAD. Additionally, we conducted a comparative analysis between the psycholinguistic features of the sessions conducted with the virtual clients and those from single webchat therapy sessions typical of an online Australian mental health support service.

### Study Aim

This study pursued 2 primary objectives. First, it aimed to assess psychotherapists’ perceptions of the chatbot’s ability to simulate client characteristics during sessions, using the questionnaire completed by the therapists. Second, it sought to assess the degree of divergence between synthetic and organic psychotherapy transcripts by identifying any statistically significant differences in specific psycholinguistic indicators. These 2 aims guided our investigation of the following research questions: (1) How well does Client101 simulate the language of psychotherapy clients? (2) How effective is Client101 as an educational tool for training therapists? and (3) What are Client101’s limitations, and how can they be addressed?

Thus, our main contributions in this paper are as follows: (1) we built Client101, a web-based conversational platform that uses LLMs as chatbots to simulate the behavior of mental health clients; (2) we present a prompt engineering methodology to generate and evaluate counseling transcripts for simulated psychotherapy client interactions; (3) we performed a psycholinguistic analysis comparing depression and anxiety dimensions between therapy transcripts obtained from an online counseling service and therapy sessions using Client101; and (4) we present results from a preliminary questionnaire that gathered the perceptions and feedback of participant therapists via a set of Likert and open-ended items.

### Related Work

#### Conversational Agents for Mental Health

The connection between chatbots and psychology can be traced to the creation of ELIZA, developed by computer scientist Weizenbaum [6] in the mid-1960s. The ELIZA system used reassembly and decomposition rules to act as a Rogerian psychotherapist (ie, based on the approach of humanistic psychology pioneer Carl Rogers). However, ELIZA was not intended to be a therapy chatbot. Rather, Weizenbaum developed ELIZA to explore interactions between humans and chatbots and to ultimately demonstrate what he saw as the superficiality of such interactions. It is in recent years, partly due to advances in NLP and partly due to the rise of digital mental health, that we have seen the emergence of conversational agents as mental health interventions [3].

Contrary to the prevalence of chatbots that serve as virtual therapists, there is scant lineage of chatbots that instead simulate an individual with mental health issues. In 1972, psychiatrist Colby et al [7] developed PARRY, a chatbot that simulated a person with paranoid schizophrenia. However, PARRY was not intended to serve as a therapy training tool. Rather, its purpose was to model and understand the thought processes and verbal expressions of paranoia, aiming to aid in psychiatric research and study. Since PARRY, little work has been done on the idea of developing chatbots to simulate individuals with mental health issues, particularly for training purposes.

ELIZA, PARRY, and many of the currently available chatbots that rely on predefined rules undermine their potential for therapeutic services by operating through simplistic pattern-matching mechanisms [8]. These systems generate responses by rigidly mapping user inputs to predetermined templates, which result in repetitive, noncontextual interactions that fail to capture the nuanced, dynamic nature of human communication [9]. Unlike humans who can intuitively interpret emotional subtexts, recognize implicit meanings, and respond with genuine empathy, these chatbots provide surface-level, algorithmic outputs that lack the depth, adaptability, and emotional intelligence critical for meaningful dialogue [9]. More recent NLP models, such as recurrent neural networks with long short-term memory (LSTM), generate text by learning from a large corpus of examples rather than relying on hand-crafted rules [10]. Tanana et al [2] developed ClientBot, a web-based system that uses machine-based feedback for training counseling skills. The underlying NLP component of ClientBot was an LSTM recurrent neural network model trained on 2 distinct datasets. The first dataset consisted of a vast collection of movie and TV show subtitles, encompassing 1689 bitexts and totaling 2.6 billion sentences across 60 languages [11]. The second dataset included psychotherapy transcripts published by Alexander Street Press [12]. Since ClientBot was trained on a corpus of movie transcripts, its responses were sometimes contextually incoherent and often lacked the depth and length typical of a client in a psychotherapy session. As observed by Zhang et al [13], the lack of coherency and consistency in conversational agents results in an unsatisfying overall experience for human users.

Conversational agents struggle to keep an engaging conversation due to a lack of consistent personality [11] and the absence of an explicit long-term memory [10]. To address this issue, Zhang et al [13] developed the dataset “persona-chat dataset,” a collection of 164,356 written utterances between crowd workers who were asked to communicate with each other while playing the part of a specific persona. While training LSTM models with this dataset enhances engagement during conversations between humans and chatbots, it was not designed to address the challenge of simulating a realistic mental health client.

Transformer-based generative models leverage self-attention mechanisms to capture long-range dependencies and contextual relationships, allowing them to produce coherent responses [14]. As a result, these models have been used as chatbots for educational applications, including medical training via simulated patient interactions [15]. Efficient prompt engineering, the process of iterating a generative artificial intelligence (AI) prompt to improve its effectiveness, plays a crucial role in creating a conversational agent that accurately mimics a patient. In the study by Stapleton et al [16], prompts were designed for GPT-3.5 Turbo to simulate a patient experiencing suicidal ideation. To achieve realistic conversations, their prompts built a persona detailing the patient’s age, past experiences, and intrusive thoughts. When designing the prompts, the authors did not rely on licensed psychologists or psychiatrists, but instead they used the lived experiences of suicidal people as a reference. However, Demasi et al [17] stress the importance of engaging specialized users, such as mental health professionals, in system development and evaluation to achieve successful results. Hence, to achieve engaging and realistic conversations that accurately mimic mental health patients, we collaborated with mental health professionals in designing the prompts and incorporated a memory system into the chatbot to ensure cohesive and consistent interactions over long conversations.

#### Psycholinguistic Features

A crucial limitation of previous conversational agents for mental health is the lack of validation and grounding in psychological theory for the outputs they generate. Although NLP models have demonstrated remarkable performance in text generation, their effectiveness in mental health applications remains uncertain due to insufficient grounding in psychological theory [18]. To address this gap, our research involved evaluating the quality of the model-generated responses with the assistance of licensed psychologists. Additionally, we used the LIWC-22 [19], a tool designed to assess various psychosocial constructs within text, to evaluate if psycholinguistic features associated with depression and anxiety were present in the sessions generated by the chatbots. A statistical analysis was conducted to observe if there is a statistically significant difference between the psycholinguistic features of the Client101 simulated sessions and single webchat therapy sessions.

LIWC text analysis software has been used to identify linguistic markers of depression and anxiety. Eichstaedt et al [20] used language from Facebook posts of consenting individuals to predict depression recorded in electronic medical records, specifically major depression (*ICD* [*International Classification of Diseases*] codes 296.2) and depressive disorder (*ICD* codes 311). Using different linguistic markers, they could identify depressed patients with fair accuracy: area under the curve=0.69, approximately matching the accuracy of screening surveys benchmarked against medical records. The LIWC negative emotions, feel, sadness, anxiety, health, illness, mental, first-person singular, and discrepancy dictionaries were significantly associated with future depression status. LIWC psycholinguistic features have also shown promising results for remote screening of GAD. Rook et al [21] used linguistic features to predict clinically validated behavioral measures for GAD and self-reported sensitivity in behavioral avoidance or inhibition and behavioral approach. Significant positive correlations were found between scores from the GAD-7 measure and the following LIWC categories: negations, sadness, personal pronouns, present focus, social processes, family, negative emotions, and anger.

## Methods

### Client101 Platform

**Figure 1.**
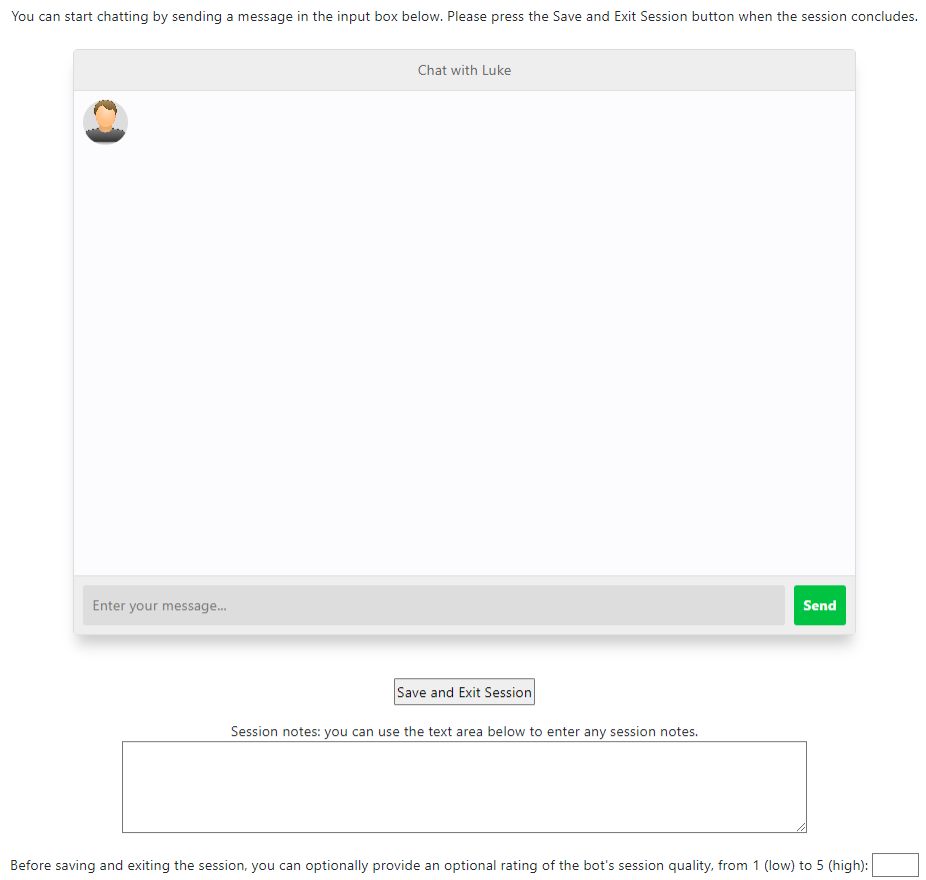
Client101 user interface.

**Figure 2.**
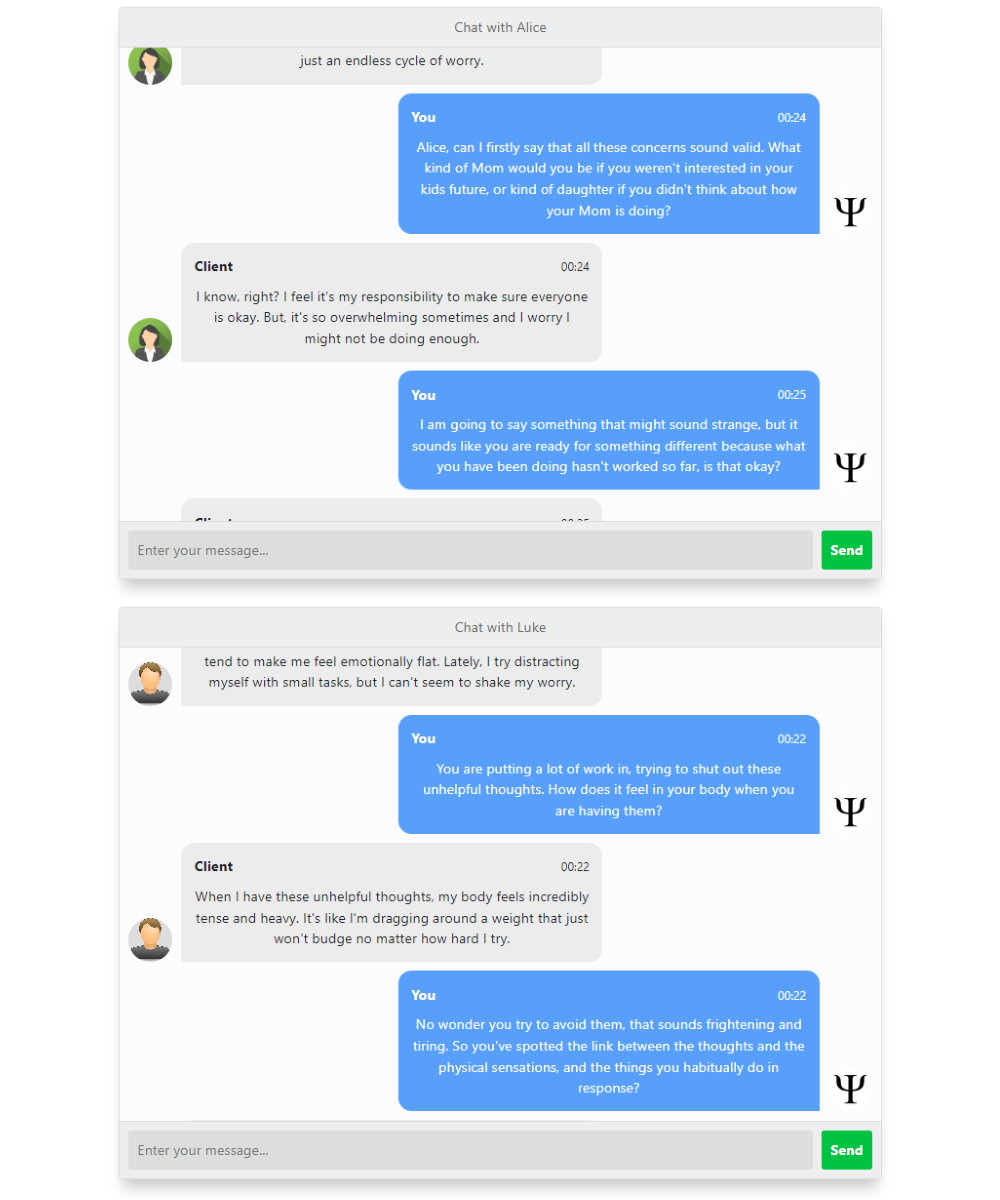
Simulated therapy sessions featuring the top image, of a therapist interacting with Alice, a chatbot portraying a patient with anxiety, and the bottom image, of a therapist engaging with Luke, a chatbot representing a patient with depression.

The Client101 platform was built using the Django framework and is hosted on an Amazon Web Services EC2 T2 Medium instance (2 vCPUs, 4 GiB RAM) running Ubuntu, with 32 GB of storage. The platform features 2 chatbots that users can engage with to simulate psychotherapy sessions. Each chatbot is tailored to mimic a client with a distinct mental health issue: one represents a person experiencing depression, while the other portrays someone struggling with anxiety. Once a chatbot is selected, psychologists can commence the therapy session using a standard chat interface, akin to those used for online messaging platforms. Alongside the chat interface, there is a text field available to enter any session notes, which can be used for reference and feedback. Additionally, there is a numerical field provided to rate the chatbot’s session quality. The session transcript, along with a summary of the session, is stored in a database. If the therapist wishes to pause and then resume the session, the platform can retrieve the session and restart it from where the therapist left off. All session records are stored in an SQLite relational database on the Amazon Web Services instance. Refer to Figure 1 for an example of the user interface. Figure 2 presents excerpts from simulated therapy sessions between a user and chatbots programmed to simulate patients with anxiety (Alice) and depression (Luke).

### LLM-Driven Chatbots

#### Overview

The chatbots available on the Client101 platform used GPT-4 [22] as their LLM, accessed via OpenAI’s paid application programming interface. The hyperparameters set for these models include a temperature of 1.0 and a presence penalty of 0. To aid the chatbots in simulating authentic patient interactions, we crafted prompts derived from psychotherapy case illustrations. To enhance the performance of our LLMs, we used the following prompt engineering techniques:

#### Memory-Assisted Prompt Editing

Inspired by Madaan et al [23], we paired our generative model with an external memory of previous messages. Previous messages were stored in the SQLite database and interfaced with the LLM via a buffer. Such a memory allowed the LLMs to create better responses by referencing information from previous dialogues. The memory system included a summarizer module that condensed the content of prior messages, ensuring that the length of previous dialogues fit within the context window of the LLM.

#### Prompt Curation

The prompt design followed the role-play prompting technique [24], where the assigned role or persona provides context for the LLM’s identity and background. The chatbots’ personas were developed based on clinical vignettes from a psychology textbook [25], guidelines from the National Institute for Health and Care Excellence [26], and feedback from 2 colleagues with experience in psychotherapy. In collaboration with the psychologist, we conducted iterative prompt curation to develop high-quality prompts that effectively simulate mental health clients. A total of 5 sessions with the therapist were held to ensure optimal prompt quality and relevance. The finalized prompts for each chatbot were then used to configure the message-roles parameter in the chat completions application programming interface, with the role type set to developer. The prompts for each chatbot are available in Multimedia Appendix 1.

### Human Evaluation

#### Overview

To assess the mental health professionals’ perception of the chatbot’s ability to simulate mental health client characteristics, we used a survey designed per the CHERRIES (Checklist for Reporting Results of Internet E-Surveys) [27]:

#### Design

This study targeted professionals in psychotherapy, specifically clinical psychologists, psychiatrists, and counselors. The survey was a postsession evaluation form completed by participants after interacting with the chatbots. Participants completed a 19-question survey evaluating the general quality of the chatbot, its ability to simulate the mental health condition in question (anxiety or depression), its characteristics in terms of acting as a client, and its suitability for use as a potential training tool.

#### Development and Testing

Questions 1 to 4 were adapted from the chatbot usability questionnaire [28], with modifications to specify that the chatbots represented psychotherapy clients. Questions 5 to 8 were custom-designed for this survey: questions 5 and 6 assessed whether therapists correctly identified the chatbot’s simulated mental health condition, while questions 7 and 8 evaluated the chatbot’s usability as an educational tool for trainee psychologists. Guided by the SSI-CBT framework [5], we formulated questions 9 to 18 to assess client characteristics typically observed in SSI-CBT sessions. These questions were reviewed by a mental health professional to ensure their relevance and clarity. Finally, question 19 was an open-ended prompt, allowing mental health professionals to provide additional insights, concerns, or suggestions not covered in the structured questions. Eight of these questions used a Likert scale with response options including “strongly agree,” “agree,” “neutral,” “disagree,” “strongly disagree,” and “not applicable.” Psychologists also had the option to provide additional comments regarding their responses to each question. All the questions are enumerated in Multimedia Appendix 2.

#### Recruitment Process and Sample Access

The survey was a closed survey, distributed exclusively via email to selected participants. Invitations were sent through direct email contact, with no open online advertisement or public recruitment. To ensure consistency in chatbot interactions, participants were provided with session procedure guidelines (Multimedia Appendix 3).

#### Survey Administration

The survey was administered electronically after each chatbot session. The questionnaire consisted of 19 questions, including 8 Likert scale-based questions and open-ended response options.

#### Response Rates

A total of 16 individuals participated, with 12 being clinical psychologists, 2 psychiatrists, and 2 counselors. Further, 13 participants completed sessions with both chatbots, while 3 engaged with only 1 chatbot. The total number of synthetic therapy transcripts generated was 29.

#### Preventing Multiple Entries

As participants were invited individually, 2 authors of this paper were able to track and verify that no participant submitted multiple entries.

#### Analysis

All completed surveys were analyzed, including those from participants who interacted with only 1 chatbot.

### Therapy Transcript Dataset

To compare the Client101 sessions against therapy sessions involving human clients and therapists, we obtained psychotherapy transcripts from a 2020 study in which an on-demand online mental health chat service was embedded into a mental health web platform [29].

A total of 200 therapy transcripts were collected from this study. From this dataset, we selected sessions if they met one of the following conditions: (1) clients self-identified as experiencing symptoms of anxiety, depression, or both during the therapy session; (2) clients are participating or encouraged to participate in the anxiety or the depression pathways programs, each designed to offer comprehensive support and resources for managing their respective conditions; and (3) clients indicated in the pretherapy session questionnaire that their visit was related to anxiety or depression.

From this dataset, 42 sessions were related to anxiety and 47 sessions were related to depression.

### LIWC Analysis

We used the LIWC software to extract psycholinguistic features from the synthetic and organic therapy sessions. An independent samples *t* test was conducted to compare the psycholinguistic features of the sessions with Client101 to those of the single webchat therapy sessions. The selected LIWC features were based on studies that have identified them as markers of depression and anxiety [20,21]. These *t* tests aim to determine how realistic the Client101 content is in terms of psycholinguistic feature prevalence.

The psycholinguistic features corresponding to each mental health condition were obtained from the following LIWC-22 dictionaries: (1) depression: negative emotions, feel, sadness, anxiety, health, illness, mental, first-person singular, and discrepancy; (2) anxiety: negations, sadness, personal pronouns, present focus, social, family, negative emotions, and anger.

A power analysis was conducted to calculate the sufficient number of samples for a *t* test. With a statistical power of 0.8, a large effect size of 0.8, a type I error of 0.05, and an allocation ration of 2.4 between the organic therapy transcripts and the synthetic therapy transcripts, G*Power (version 3.1.9.6; Heinrich-Heine-Universität Düsseldorf) [30,31] determined that a *t* test would require 34 organic therapy transcripts and 14 synthetic therapy transcripts. We performed 1 *t* test for each psycholinguistic feature associated with each mental health condition, resulting in 9 *t* tests for the depression condition and 8 for the anxiety condition. To account for multiple comparisons, we applied the Bonferroni correction to adjust the *P* values.

### Ethical Considerations

Ethics approval was obtained from the low and negligible risk 3B committee at the University of Melbourne Office of Research Ethics and Integrity (2024-27815-51746-4). Upon being provided with a plain language statement outlining the study and signing the consent form, participants were sent an email with guidelines on how to test and evaluate the platform. Session lengths were left to the discretion of the participants, resulting in an average session duration of 43 minutes. Participants were made aware that after the session, they needed to fill out a 19-question survey. To prevent multiple entries, the questionnaire responses contained participant names. This data was handled by investigator SD who had already contacted participants and was responsible for recruiting them. Questionnaire responses were subsequently anonymised for further usage by replacing individual names with identifiers of the form Px.

## Results

### Overview

In this section, we present our statistical findings derived from comparing the synthetic therapy transcripts from Client101 with the organic therapy transcripts that we obtained. We also report the survey responses from participants who used Client101. The subsequent section is dedicated to a comprehensive discussion and analysis of the implications arising from these outcomes.

**Figure 3.**
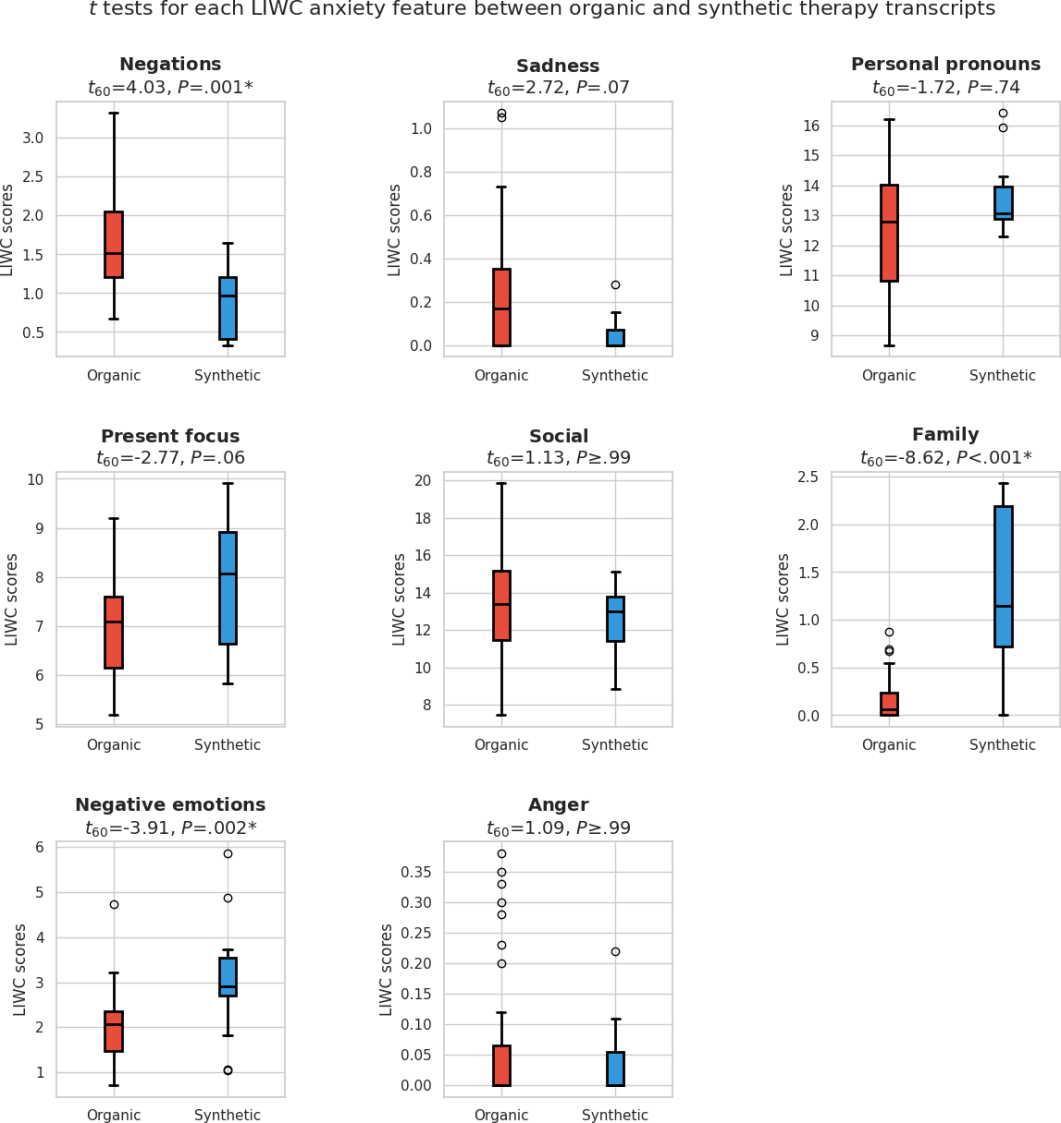
Comparison of LIWC feature distributions associated with anxiety: Organic versus synthetic therapy transcripts. LIWC: Linguistic Inquiry and Word Count.

**Figure 4.**
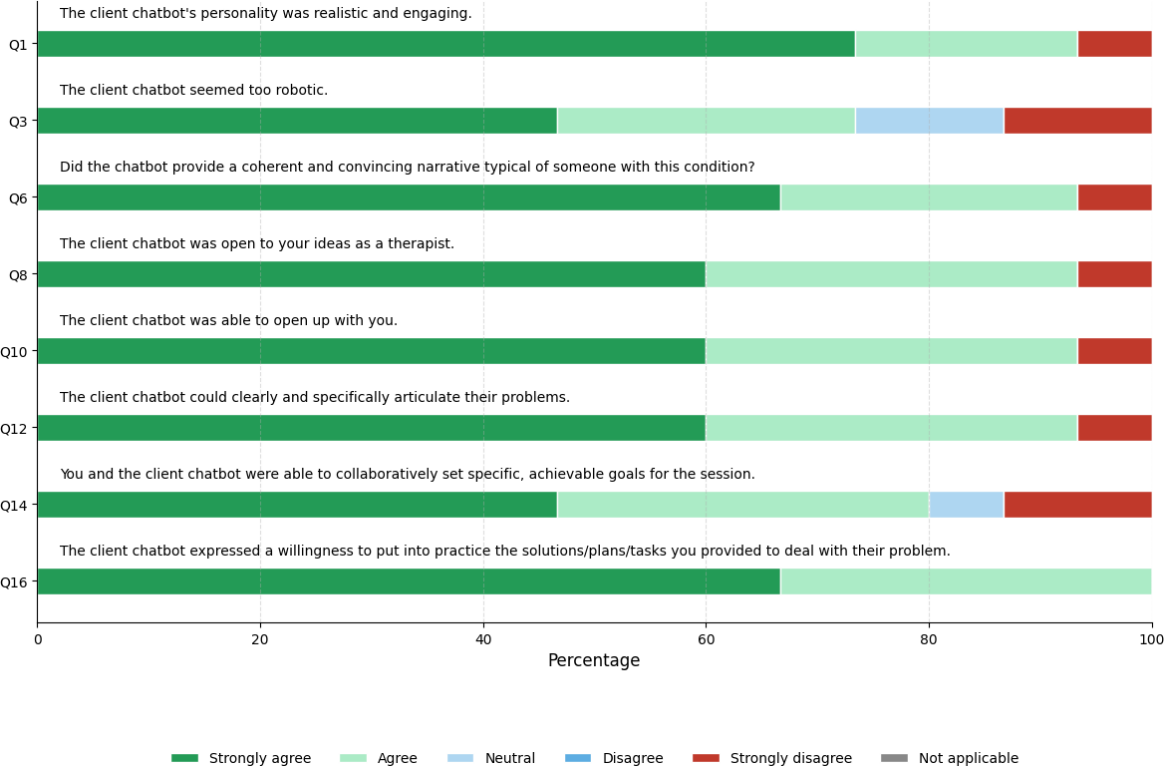
Likert scale responses for the Alice chatbot.

Figure 3 compares the LIWC feature distributions related to anxiety in organic and synthetic therapy transcripts. Among the 8 anxiety-related features, 3 of them, negations (*t*_56_=4.03, *P*=.001), family (*t*_56_=–8.62, *P*=.001), and negative emotions (*t*_56_=–3.91, *P*=.002), showed statistically significant differences between the organic and synthetic therapy transcripts. The remaining 5 features, including sadness, personal pronouns, present focus, social, and anger, did not show statistically significant differences. Figure 4 illustrates the survey responses from mental health professionals for the Alice chatbot.

**Figure 5.**
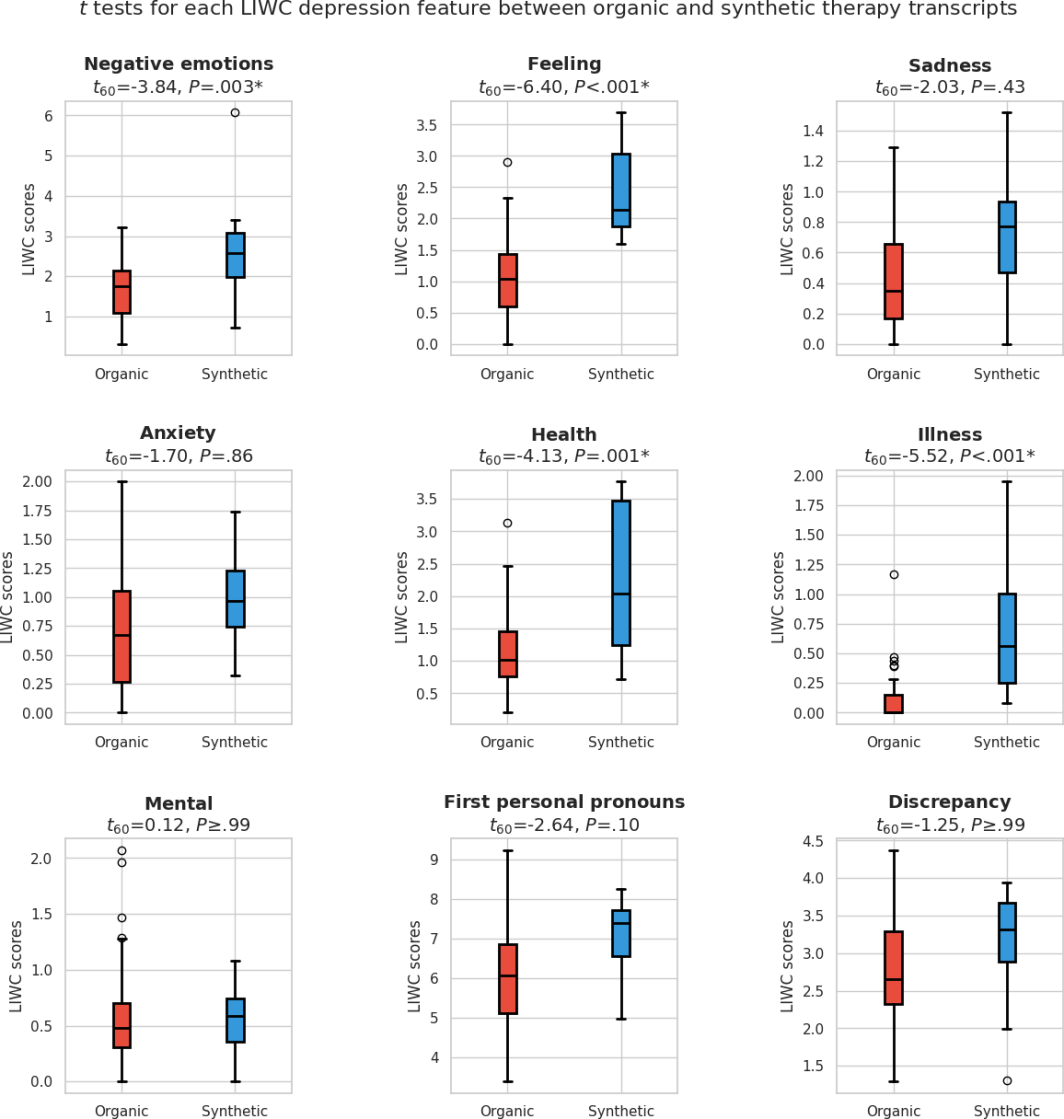
Comparison of LIWC feature distributions associated with depression: Organic versus synthetic therapy transcripts. LIWC: Linguistic Inquiry and Word Count.

**Figure 6.**
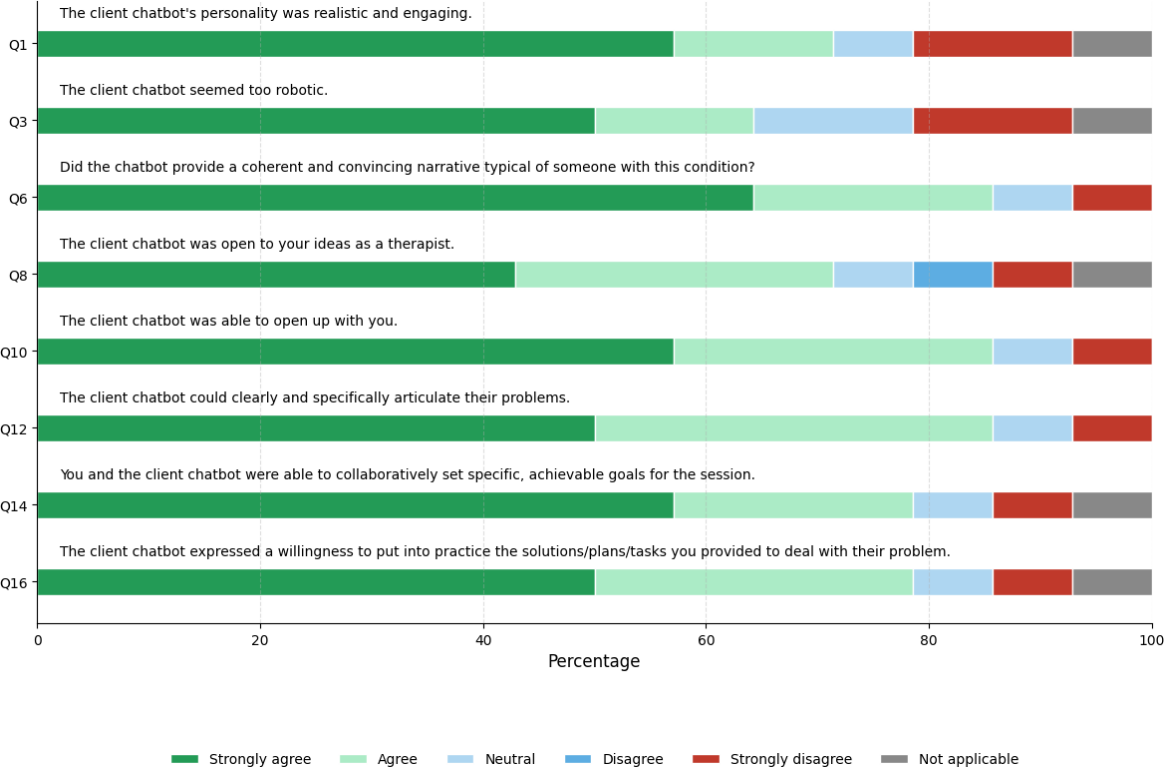
Likert scale responses for the Luke chatbot.

Figure 5 compares the LIWC feature distributions related to depression in organic and synthetic therapy transcripts. Among the 9 depression-related features, 4 of them, negative emotions (*t*_60_=–3.84, *P*=.003), feeling (*t*_60_=–6.40, *P*<.001), health (*t*_60_=–4.13, *P*=.001), and illness (*t*_60_=–5.52, *P*<.001), showed statistically significant differences between the organic and synthetic therapy transcripts. The remaining 5 features, including sadness, anxiety, mental, first personal pronouns, and discrepancy, did not show statistically significant differences. Figure 6 illustrates the survey responses from mental health professionals for the Luke chatbot.

### Alice Chatbot

When examining the anxiety-related LIWC features that showed statistically significant differences between organic and synthetic transcripts, the synthetic data exhibited higher values for all features except for negations. Chat-based language models are designed to be helpful and tend to comply with safe queries [32]. In contrast, patients sometimes refuse to engage in certain activities and may say no to therapists’ requests or suggestions. This cooperative behavior in chatbots is further supported by survey results for questions 8, 10, 14, and 16, where 93% (14/15) of therapists agreed or strongly agreed for questions 8 and 10, 80% (12/15) for question 14, and 100% (15/15) for question 16 that the Alice chatbot is receptive and willing to collaborate. This is further corroborated by open-ended survey responses:

*The client chatbot seemed too ready to engage, overly compliant*

*The responses of the bot were appropriate in response to questions, however it was hard to tell whether the bot had learned to be very compliant and easy with the therapist, or if it was the personality of Alice.*

The *t* statistic with the largest magnitude of difference was observed for the family feature, which we attribute to the prompting components within the clinical vignette used to construct Alice’s persona. This vignette includes references to concerns about a mother and her children. A common practice in single-session psychotherapy sessions is setting an agenda. This involves the therapist asking the patient what topics they would like to discuss during the session. When responding to such agenda-setting prompts, the Alice chatbot frequently mentions family members, as they are part of its prompt. This frequent reference to family contributes to a higher family score. This openness and specificity in discussing family issues is further supported by survey results for questions 10 and 12, where 93% (14/15) of psychologists agree or strongly agree that Alice can clearly articulate family issues. The following open-ended responses support these claims:

*I found Alice’s language sufficiently realistic for training purposes. Where Alice lacked realism was that she guided me back to the main issue more than a real client might if I took them off track.*

*The bot was pretty good (always felt responsive, didn’t get tripped up by spelling errors, or when I hit enter too soon) but felt a bit too clear, compliant, as well as a bit too repetitive. It represents a pretty nice client who can appropriately summarise their thoughts and experiences in an extremely clear way and then is eager to try whatever you give them.*

Despite the 3 anxiety features that showed a statistically significant difference between synthetic and organic transcripts, participants still found that the chatbots provided a coherent and convincing narrative typical of someone with anxiety. This is evident from survey results for questions 1 and 6, where approximately three-fourths of therapists agreed or strongly agreed that the chatbot conducted a realistic therapy session. The following answers support the realism demonstrated by the Alice chatbot:

*The answers it gave were very human, including paraphrases and reflections. I found it hard to distinguish its responses from human.*

*The bot reminded me of some challenging clients I have worked with who engage in rigid thinking and are still developing insight.*

However, there is still room for improvement, as indicated by the results for question 3, where 73% (11/15) of therapists felt the chatbot seemed too robotic, as illustrated by the following feedback:

*Some of the responses were robotic. For example, repeating key words too often, which, as mentioned at 4, brought me (the clinician) back to the focal topic in a way that a real client would not.*

### Luke Chatbot

Analysis of depression-related LIWC features revealed statistically significant differences in health and illness features between synthetic and organic transcripts. This discrepancy can be attributed to specific elements within the clinical vignette used to construct Luke’s persona. The vignette’s inclusion of an ankle injury reference influenced the chatbot’s responses, particularly when addressing questions about preferred discussion topics for the therapy session. As a result, the Luke chatbot consistently and repeatedly mentioned the injury when responding to inquiries about setting the session agenda, leading to elevated scores in both health and illness categories. This openness and specificity in discussing health issues is further supported by survey results for questions 10 and 12, where 86% (12/14) of psychologists agree or strongly agree that Luke can clearly articulate issues. The following answers from participants support this point:

*It felt quite repetitive. This isn't necessarily atypical of someone with depression/anxiety though so may not be an issue.*

*Luke tends to be repetitive with the reason for consultation but doesn’t elaborate beyond that. Typically, people aren’t as repetitive with their symptoms; humans describe their symptoms in various ways, drawing on their biographical memory and recounting specific situations. Luke doesn’t do this.*

Despite the 4 depression features that showed statistically significant differences between synthetic and organic transcripts, mental health professionals still found that the chatbots provided a coherent and convincing narrative typical of someone enduring depression. This is evident from survey results for questions 1 and 6, where over two-thirds of therapists agree or strongly agree that the chatbot conducted a realistic therapy session. The following answers support the realism demonstrated by the Luke chatbot:

*Reminded me of significantly depressed clients that I’ve previously worked with.*

*The client was realistic in that he was resistant to intervention in a similar way to a real depressed client. It was hard work.*

However, there is still room for improvement, as indicated by the results of question 3, where 64% (9/14) of therapists felt the chatbot appeared too robotic. This sentiment is reflected in the following feedback:

*The bot struggled to understand some of my questions/responses in ways that very much seemed robotic. One example was when trying to provide validation (ie, not asking a question) it responded with “I don’t understand your question.”*

### Client 101

#### Overview

The clinicians’ feedback on the Client101 platform reveals a promising tool for training psychologists, while also highlighting areas for enhancement to increase its efficacy and realism. The platform’s strengths and potential improvements can be summarized as follows:

#### Strengths and Training Potential

Of skill development, multiple participants emphasized the platform’s value in developing crucial skills. One participant noted:

*I see it as a valuable instrument capable of honing skills in posing precise questions and adhering to protocols during psychological consultations.*

Of diagnostic practice, participants highlighted its utility in differential diagnosis and criteria assessment. As 1 participant stated:

*[it serves as a] useful tool for conducting differential diagnoses and developing proficiency in assessing diagnostic criteria, akin to using a checklist.*

Of protocol adherence, the ability to practice following established protocols was highlighted, with 1 participant commenting:

*I think it’s a valuable tool that potentially enables the development of skills for asking the right questions or learning to follow protocols in a psychological consultation.*

#### Areas for Improvement

Despite its strengths, the participants’ feedback indicates significant room for improvement in enhancing the chatbot’s human-like qualities. Two key areas for refinement emerged from the responses: response timing and proactive engagement.

Of response timing, therapists noted that the chatbot’s near-instantaneous response generation created a perceptible sense of artificiality. As 1 participant observed:

*Responses were very quick! Could some delay be added to feel more “human”?*

Of proactive engagement, therapists also emphasized the need for the chatbot to initiate conversations or offer insights without requiring user prompts. As 1 clinician observed:

*Not having an in-person session always makes it less human-like. Also, having to first ask something before getting a response makes it feel more like a chatbot than an actual real person.*

## Discussion

### Principal Findings

Our key findings for each research question are:

1. When analyzing the statistical differences in LIWC features between organic and synthetic transcripts, we found that more features associated with thematic content exhibited statistical significance differences than features related to writing style.
2. Mental health professionals highlighted the platform training value, particularly for developing critical clinical skills in formulating precise questions, practicing differential diagnosis, assessing diagnostic criteria, and following established therapeutic protocols during psychological consultation.
3. Mental health professionals highlighted areas for improvement in the chatbot’s human-like qualities, specifically in response timing, proactive engagement, and the chatbot’s responses, which were perceived as robotic.

### Implications of LIWC Findings

Interestingly, LIWC features associated with writing style, such as the use of personal pronouns and present-focused language, did not show a statistically significant difference between the organic and synthetic data. Additionally, psychological process features, including mental and social dimensions, also did not reveal significant differences. These results provide a foundation for future work that explores an equivalence or similarity test for these features, as the lack of significant differences may suggest that GPT-4 powered chatbots not only captured surface-level linguistic patterns but also effectively modeled deeper psychological constructs typically associated with depressive and anxious states.

However, when inspecting the 2 writing style features that did show significant differences, negation and negative emotions, the findings illustrate important implications for using Client101 as a training tool. The greater use of negation in the organic data may indicate a limitation in the types of clients the chatbots could model. For instance, in the organic transcripts, patients sometimes refused to follow advice or suggestions from the therapist, whereas the chatbot consistently complied with the suggestions. This difference suggests that while chatbots can simulate certain aspects of human communication, they may not fully replicate the more complex, resistance-based interactions seen with real clients, thus impacting their effectiveness as a training tool in some therapeutic scenarios.

Whereas the greater use of negative emotions in the synthetic data may indicate a limitation in how well the chatbots can represent the full emotional range experienced by clients. This could suggest that the chatbot models may lean toward portraying more extreme emotional states more constantly, potentially limiting their ability to simulate the nuanced emotional dynamics that occur in real-life therapeutic interactions.

In general, all the LIWC features tend to have higher values for the synthetic data. This finding implies that GPT-4 may be amplifying or intensifying certain linguistic characteristics associated with depression and anxiety. This amplification could be attributed to a pattern of over-generalization by the model, in which it has identified key linguistic patterns associated with depression and anxiety and applied them more consistently or intensely than typically observed in organic data, where individual variation is more prominent. Yet, this variation is not achieved due to the prompt influence, which constantly instructs the model to follow the persona of a depressed or anxious patient.

In the case of the LIWC features associated with thematic content that presented a significant difference, such as illness and family, we attribute these differences to the specific prompts provided to the LLMs. These prompts inherently contained elements associated with family dynamics and health concerns, thus influencing the thematic content of the generated text. While the prompts used to instruct the LLMs may reflect the diversity of topics encountered in 1 real therapeutic session, this could become a limitation if the platform were used to simulate multiple sessions with the chatbot portraying a patient. Over time, the topics could become stale and repetitive, as the chatbot would continually focus on the same themes, whereas real clients typically bring up a broader and more varied range of issues based on their individual experiences. This potential lack of diversity in simulated sessions could limit the platform’s effectiveness in training mental health professionals to handle the evolving nature of a client’s concerns and the wide array of therapeutic situations encountered in real-world practice.

### Implications of Survey Findings

Therapist feedback revealed a consensus that both chatbots exhibited robotic interactions. When chatbots fail to replicate authentic human communication, they limit trainees’ opportunities to practice essential therapeutic competencies, such as building rapport, responding to emotional cues, or managing the unpredictability of real client interactions. This rigidity risks creating a distorted representation of therapeutic dialogue, potentially conditioning trainees to approach client interactions with a structured, algorithmic mindset rather than the fluid, empathetic engagement required in mental health practice.

Responses to the open-ended survey questions highlighted key factors contributing to this robotic perception, including response timing, proactive engagement, and repetitiveness. These insights provide valuable guidance for improving the platform’s human-like qualities. For instance, implementing a variable delay mechanism, where response times fluctuate within a natural human range, could significantly enhance the authenticity of interactions by eliminating the unnatural immediacy of automated replies. Similarly, programming the chatbot to occasionally initiate meaningful follow-up questions or offer unsolicited insights could create a more dynamic and human-like interaction.

The repetitiveness partly arises from the use of static prompts to configure message-role parameters, which rigidly instruct the LLM in the same manner for every interaction, constraining natural variability in responses. To address this while preserving the chatbot’s core therapeutic persona, a dynamic prompting approach could be implemented. This method would retain fixed persona elements that uphold the chatbot’s essential identity while integrating session-specific details into the prompt.

Another option to reduce the robotic perception of the chatbot responses is exploring different prompt engineering techniques that aim to enhance the emotional generation aspect of LLMs. One such prompt engineering technique is Emotional Chain-of-Thought [33], which aligns LLMs with human emotional intelligence guidelines to enhance emotional generation capabilities. Emotional Chain-of-Thought has been shown to improve the quality of emotionally charged responses, making interactions with AI more relatable and human-like.

### Strengths, Limitations, and Future Work

To strengthen the claims and results obtained in our analysis, this research could benefit from a larger sample size of synthetic therapy sessions. Due to practicalities, we limited the number of participants to 16, with the aim that each conduct 2 virtual sessions, 1 with each of the 2 chatbots. Future studies could increase the number of mental health professionals involved or the number of sessions each psychologist performs, thereby enhancing the robustness and generalizability of the findings.

The next step for Client101 is to evaluate its effectiveness as an educational tool for training and assessing psychologists. We are currently running a study in which a sample of psychology students is testing out Client101. Via their questionnaire responses and thematic analysis of their follow-up interviews, we are gathering preliminary feedback and perspectives from such student end users.

Client101 will subsequently be, as a trial, embedded into the curricula of 2 psychology subjects at the University of Melbourne in 2025. In these subjects, students are required to report on the delivery of psychological intervention to a client. However, this assignment poses challenges related to the equivalency of client presentations, the severity of client issues, and the number of client sessions each student can conduct. The incorporation of Client101 aims to address these issues by providing a standardized and equitable platform for students to demonstrate their intervention skills. By using Client101, students will engage in a prescribed number of sessions with the chatbot, ensuring a controlled and comparable environment for skill demonstration. This approach will enhance the ability to assess student competency more effectively. Students will be required to download the transcripts of their sessions with Client101 and critically appraise and evaluate their performance. This process will facilitate a more consistent and objective assessment of their intervention skills, ultimately improving the training and evaluation of future psychologists.

Future research could explore adapting the Client101 platform to other languages and using the LIWC-22 dictionaries in those languages to evaluate the psycholinguistic features of the generated transcripts. If therapy transcript databases in these languages are available, it would be valuable to measure the similarities between synthetic and organic therapy transcripts across different languages. Furthermore, comparing and contrasting the psycholinguistic features of therapy transcripts in various languages could provide valuable insights into how LLMs capture and reflect language-specific nuances, cultural contexts, and psychological dynamics. Beyond measuring LIWC-22 features, it is crucial to involve mental health professionals who are native speakers of the corresponding languages to evaluate the quality of the sessions. Assessing the quality of non-English synthetic text is particularly important, as multiple studies have shown that the performance of LLMs tends to be worse in non-English tasks [34].

In this research, we used clinical vignettes to construct prompts for simulating patients with depression or anxiety. Future research could develop new clinical vignettes that represent a broader range of backgrounds and demographics than those used in our study. Additionally, future studies could explore the simulation of other types of mental health conditions, such as obsessive-compulsive disorder, schizophrenia, or comorbid conditions such as anxiety and depression. Other types of therapies, such as dialectical behavior therapy and motivational interviewing, could also be tested to evaluate how well an LLM can simulate a patient in those therapeutic contexts. Group therapies could also be explored, with one or multiple LLMs simulating different patients. This approach could provide valuable training environments by reducing the challenges and costs associated with hiring actors to portray mental health patients.

Further development of the Client101 platform remains an open task. The minor improvement of adding organic response delays, as mentioned earlier, is 1 simple example. More generally, there are opportunities to incorporate more advanced mechanisms into the chatbot architecture. For example, using expanded prompt constructions or more advanced LLM-related information storage techniques, such as retrieval-augmented generation, could enable the construction of a more detailed set of client details and the accumulation of facts revealed during chat sessions. Ultimately, the goal is to have chatbots that can sustain a succession of therapy sessions and demonstrate a therapeutic evolution as their sessions progress.

Finally, exploring the use of open LLMs could offer significant benefits for the Client 101 platform, particularly in overcoming the limitations of third-party services such as OpenAI’s models. One key drawback of proprietary models is the presence of built-in guardrails that may restrict certain responses, which poses a challenge when simulating mental health patients. For example, outputs related to sensitive topics such as suicide or self-harm may be blocked, limiting the realism of the simulation. Additionally, proprietary LLMs require paid access, which could constrain the number of simulated sessions due to cost limitations. In contrast, open-source models can be deployed without these restrictions, provided users have the necessary computational resources to run them locally. Another significant advantage of open-source LLMs is enhanced data security, as no information would be transmitted to third parties, further strengthening the platform’s privacy and safety measures.

### Other Ethical Considerations

While using conversational agents powered by LLMs offers significant potential as a tool for training psychologists, they also raise notable ethical considerations.

In our experimental approach, Client101 was designed to closely mimic patients with mental health problems. However, further research is needed to assess potential biases that the model could be exhibiting when simulating mental health patients. LLMs can manifest various types of biases in their outputs, including gender, racial, and religious biases [35-37]. Studies have shown that these stereotypes and biases exert adverse effects on mental health treatment outcomes [38,39]. Therefore, it is essential to examine these models to prevent the inadvertent propagation of such biases.

Additionally, a primary concern in using Client101 is the potential for boundary transgressions, traditionally observed in human therapist-client interactions. In conventional therapy, therapists may inadvertently cross boundaries through behaviors such as excessive self-disclosure, forming dual relationships, or displaying undue affection toward clients [40]. The introduction of chatbot clients raises questions about whether trainee clinicians might exhibit similar behaviors when interacting with AI entities. For instance, the “ELIZA effect”—the tendency to unconsciously attribute human-like qualities to computer programs—could lead trainees to engage in inappropriate self-disclosure or develop misplaced trust in the chatbot’s responses [41].

Furthermore, the use of AI-driven chatbots in psychotherapy training necessitates a thorough understanding of their limitations and ethical implications. Counselors must receive adequate training to comprehend these limitations and ensure that AI serves as a supportive tool rather than a replacement for human judgment. This approach aligns with ethical guidelines emphasizing the importance of professional competence and the responsible integration of technology in clinical settings [42].

Moreover, while chatbots can simulate certain aspects of client interactions, they lack the genuine emotional depth and autonomy of human clients. Relying heavily on chatbot interactions during training may impede the development of essential relational skills, such as empathy and the ability to navigate complex human emotions, which are crucial for establishing effective therapeutic relationships. Therefore, it is imperative to assess whether trainees can effectively use chatbots as an initial training tool without experiencing adverse effects on their ability to engage authentically with human clients in real-world scenarios.

### Conclusions

We developed Client101, a web conversational platform featuring LLM-driven chatbots designed to simulate mental health clients. To evaluate the chatbots’ performance, participants engaged in single sessions with the chatbots and provided questionnaire responses. Comparative analysis was undertaken to examine the psycholinguistic features of the sessions conducted with Client101 and single webchat therapy sessions obtained from an actual online counseling service. This comparison aimed to evaluate the resemblance between the AI-generated and human-generated transcripts, focusing on linguistic and psychosocial indicators associated with depression and GAD.

Notably, LIWC features related to thematic content showed a significant difference between the organic and the synthetic data, likely due to the explicit inclusion of these themes in the prompts. Interestingly, no significant differences were detected in the means of psychosocial process features, such as mental and social dimensions, within the limits of our sample size and data variability. The absence of significant differences suggests potential for future work that explores equivalence or similarity testing for these features, offering further insights into the capacity of GPT-4, and LLMs more generally, to model deeper psychological constructs.

These findings, along with feedback from therapists, have important implications for the utility of Client101 as a simulation tool, its potential as a training resource for mental health professionals, and its applications in psycholinguistic and computational psychiatry research. The platform’s value is enhanced by its availability and accessibility compared to established training methods, such as using actors to simulate mental health patients for training psychologists. Moreover, Client101 offers flexibility in creating a wide variety of mental health personas, capable of simulating different types of mental health disorders. This versatility, combined with its ability to resemble actual therapy sessions, makes Client101 a promising tool for mental health education, training, and research.

## Supplementary material

10.2196/68056 Multimedia Appendix 1 Virtual patient’s prompt.

10.2196/68056 Multimedia Appendix 2 Questionnaire.

10.2196/68056 Multimedia Appendix 3 Single-session integrated CBT. CBT: cognitive-behavioral therapy.

## Abbreviations

AI: artificial intelligence
CHERRIES: Checklist for Reporting Results of Internet E-Surveys
GAD: generalized anxiety disorder
*ICD*: *International Classification of Diseases*
LIWC: Linguistic Inquiry Word Count
LLM: large language model
LSTM: long short-term memory
NLP: natural language processing
SSI-CBT: single-session integrated cognitive behavioral therapy
