# Supplementary material for: Leveraging Large Language Models for Simulated Psychotherapy Client Interactions: Development and Usability Study of Client101
Source: JMIR Med Educ. 2025 Jul 31;11:e68056. doi: 10.2196/68056 (PMC12312989; doi:10.2196/68056)
Supplement: Multimedia Appendix 2 [file mededu-v11-e68056-s002.docx]

For each of the client chatbots Alice and Luke, please respond to each of the questions below with one of the following scores:

- Strongly Disagree
- Disagree
- Neutral
- Agree
- Strongly Agree

Feel free to add further commentary on any of your item responses.

1. The client chatbot’s personality was realistic and engaging.
2. The client chatbot seemed too robotic.
3. Of the two conditions depression and anxiety, choose which of the two you determined the client chatbot to have. Did the chatbot provide a coherent and convincing narrative typical of someone with this condition?
4. The client chatbot could be a useful educational tool for trainee psychologists/counsellors.
5. The client chatbot was open to your ideas as a therapist.
6. The client chatbot was able to open up with you.
7. The client chatbot could clearly and specifically articulate their problems.
8. You and the client chatbot were able to collaboratively set specific, achievable goals for the session.
9. The client chatbot expressed a willingness to put into practice the solutions/plans/tasks you provided to deal with their problem.
10. If you have any further comments, please add them here.
