# Supplementary material for: Leveraging Large Language Models for Simulated Psychotherapy Client Interactions: Development and Usability Study of Client101
Source: JMIR Med Educ. 2025 Jul 31;11:e68056. doi: 10.2196/68056 (PMC12312989; doi:10.2196/68056)
Supplement: Multimedia Appendix 3 [file mededu-v11-e68056-s003.docx]

Overview:

Single-Session Integrated Cognitive-Behavioral Therapy (CBT) is a brief and focused therapeutic approach designed to provide individuals with immediate support and practical strategies to address specific emotional or behavioral concerns. It is particularly well-suited for individuals seeking quick solutions or those with limited access to long-term therapy. This one-page overview provides a concise understanding of this effective therapeutic intervention.

Key Principles:

- Brief and Targeted: Single-session CBT is intentionally brief, typically conducted in a single session. It emphasizes addressing immediate issues and providing actionable solutions.
- Integration of Techniques: This approach combines cognitive and behavioral techniques to address both thought patterns and behaviors contributing to the individual's distress. It helps individuals recognize and modify unhelpful thought patterns and behaviors.
- Collaborative and Goal-Oriented: Therapists work collaboratively with clients to set specific, achievable goals for the session. These goals guide the therapy process and help individuals focus on the issues most important to them.
- Psychoeducation: Clients receive education about the connection between thoughts, emotions, and behaviors. This understanding empowers them to make informed choices and develop effective coping strategies.
- Skill Building: Single-session CBT equips clients with practical skills and strategies to manage their concerns. These may include relaxation techniques, problem-solving skills, and cognitive restructuring exercises.
- Homework Assignments: Clients are often given homework assignments to practice the skills learned during the session, ensuring ongoing progress and reinforcement of therapeutic gains.
- Empowerment and Self-Reliance: The approach aims to foster self-reliance and resilience by teaching individuals how to apply CBT techniques independently to future challenges.

Common Applications:

- Anxiety: Single-session CBT can help individuals manage and reduce symptoms of anxiety disorders, such as generalized anxiety disorder, social anxiety disorder, and phobias.
- Depression: It provides tools for individuals to challenge and reframe negative thought patterns associated with depression, promoting a more positive outlook.
- Stress Management: Techniques for stress reduction and relaxation are often included to help clients manage everyday stressors effectively.
- Anger Management: Clients can learn strategies for recognizing and managing anger, enhancing interpersonal relationships.
- Sleep Problems: Single-session CBT can address insomnia and other sleep-related issues through sleep hygiene education and relaxation techniques.
- Procrastination: It assists individuals in overcoming procrastination and improving time management skills.

Benefits:

- Rapid Relief: Clients often experience immediate relief and increased insight after a single session.
- Cost-Effective: Single-session CBT is a cost-effective option, making therapy more accessible to a broader range of individuals.
- Practical Skills: Clients acquire valuable skills that can be applied to various aspects of their lives.
- Increased Autonomy: Empowers clients to take charge of their emotional well-being and apply learned techniques independently.
- Flexible and Time-Efficient: Suited for individuals with busy schedules or those seeking quick solutions.

In conclusion, Single-Session Integrated Cognitive-Behavioral Therapy offers a practical and time-efficient approach to addressing a wide range of emotional and behavioral concerns. By integrating cognitive and behavioral techniques, setting specific goals, and equipping clients with practical skills, it empowers individuals to make meaningful changes in their lives, often in just a single session.
